# Design of nanoscaled heterojunctions in precursor-derived *t*-ZrO_2_/SiOC(N) nanocomposites: *Transgressing the boundaries of catalytic activity from UV to visible light*

**DOI:** 10.1038/s41598-019-57394-8

**Published:** 2020-01-16

**Authors:** Shakthipriya Bhaskar, Eranezhuth Wasan Awin, K. C. Hari Kumar, Abhijeet Lale, Samuel Bernard, Ravi Kumar

**Affiliations:** 1 Laboratory for High Performance Ceramics, Department of Metallurgical and Materials Engineering, Indian Institute of Technology Madras (IIT Madras), Chennai, 600036 India; 2 Univ. Limoges, CNRS, IRCER, UMR 7315, F-87000 Limoges, France

**Keywords:** Photocatalysis, Synthesis and processing

## Abstract

In this work, nanocomposites made of nanosized zirconia crystallized *in situ* in an amorphous silicon oxycarbo(nitride) (SiOC(N)) matrix have been designed through a precursor route for visible light photocatalytic applications. The relative volume fraction of the starting precursors and the pyrolysis temperatures not only influences the phase fraction of zirconia crystallites but also stabilizes the tetragonal crystal structure of zirconia (*t*-ZrO_2_) at room temperature. The presence of carbon in interstitial sites of zirconia and oxygen vacancy defects led to drastic reduction in the band gap (2.2 eV) of the nanocomposite. Apart from being a perfect host avoiding sintering of the active phase and providing mechanical stability, the amorphous matrix also reduces the recombination rate by forming heterojunctions with *t*-ZrO_2_. The reduction in band gap as well as the formation of heterojunctions aids in harnessing the visible light for photocatalytic activity.

## Introduction

Large scale industrialization and urbanization has led to extensive pollution and contamination of rivers and water bodies, posing a threat to future generations and sustainable development^1^. Chemical industries, leather factories, paint manufactures, dyeing and plating units release harmful untreated waste directly into our water bodies^2–4^. These are non-biodegradable and toxic to humans as well as flora and fauna. Filtration with activated carbon, coagulation using chemical agents, ozonization, flocculation and reverse osmosis are some of the techniques that are frequently used to remove these harmful substances^5–8^. However, these techniques are not effective in complete removal and merely result in the transfer of chemicals from one source to another. The photocatalytic phenomenon is capable of converting these toxic substances (EDTA, Cr(VI), nitrite) to harmless counterparts such as hydrogen, Cr(III) and nitrates^9–11^. This is achieved *via* a catalyst that upon subsequent excitation produces electrons and holes. These electrons and holes produce superoxide and hydroxyl radicals that are responsible for the oxidation and reduction processes^12–15^. But the efficiency of photocatalyst is largely dependent on the recombination rate of these electrons and holes. This drives the need for developing suitable catalysts which could be eventually scaled up for a large-scale clean-up drive^16–19^.

The wide band gap (3.25–5.1 eV) combined with a negative conduction band potential (−1.0 V vs. NHE at pH 0)^11^ has recently spurred a lot of research on zirconia as a photocatalyst^20–23^. It is important to note that the band gap value depends on the crystal structure (3.84 eV for cubic, 4.11 eV for tetragonal and 4.51 eV for monoclinic)^24^, defects and processing route of the ceramics^25^. From the available limited literature, it is known that the presence of both monoclinic and tetragonal phases of zirconia^26–28^ is beneficial for photocatalysis. The high temperature tetragonal phase of zirconia namely *t*-ZrO_2_ can be stabilized at room temperature by using suitable dopants like CaO, MgO and Y_2_O_3_ or by reducing the crystallite size^24^. It is well established that nanostructured materials with large surface area greatly enhance the absorption of the irradiated light during photocatalysis^29^ and hence it appears advantageous to synthesize zirconia with crystallite size restricted to nanoscale dimensions for photocatalytic applications.

Anodization^26,27^, electric arc discharge^28^ and combustion method^30^ are some of the common routes to synthesize nano-zirconia. However, the tedious, time-consuming processing steps, agglomeration and non-uniform size distribution, instability of the metastable tetragonal phase and its subsequent transformation to a pure monoclinic structure^26^ are some of the major drawbacks of these synthesis routes. Furthermore, the majority of research has been concentrated on UV light degradation of zirconia (bandgap values in the range of 4.71–5.0 eV and 3.1–3.54 eV have been reported)^9,21,26–28,30^.

Recently, black ZrO_2_ particles with very low bandgap (~1.5 eV) were reported to be photocatalytically active in the visible range and this was attributed to the presence of surface defects^31^. Doping of zirconia with carbon and nitrogen is also seen to reduce the band gap and increase the absorption of visible light^32–34^. Researchers have reported band gap values of 2.37 and 3.8 eV for carbon and nitrogen doped zirconia respectively^32,34^. It is clear that the photocatalytic response of ZrO_2_ is governed to a large extent by the crystal structure, crystallite size, specific surface area, surface adsorbed species, surface defects and chemical composition of zirconia. This necessitates the need for developing a simplified processing approach wherein control of a large number of factors which contribute to the photocatalytic effect in the visible region is possible.

Dire *et al*.^35^ have synthesized zirconia crystallites in an amorphous silicon oxycarbide (SiOC) matrix through a sol-gel route using diethoxydimethylsilane as SiOC precursor and zirconium n-propoxide as ZrO_2_ precursor. Ionescu *et al*.^36^ have prepared ZrO_2_/SiOC ceramic nanocomposites through both *in-situ* addition of zirconium tetra(n-propoxide) as precursor and *ex-situ* addition of zirconia nano particles to polymethylsilsesquioxane as a matrix precursor (=preceramic polymer). Ceramics synthesized through the *ex-situ* addition of zirconia showed better crystallization resistance of matrix. This could be attributed to the higher zirconia content obtained through the *ex-situ* process. Also, the residual free carbon content was lower in *ex-situ* process than *in-situ*. Both of them clearly indicate that with the increase in the zirconia content there is great enhancement in the properties of matrix. In both cases, the photocatalytic behavior of nanocomposites was not investigated.

In this work we report the photocatalytic behavior of *t*-ZrO_2_/SiOCN nanocomposites with *in-situ* formation of crystallized zirconia stabilized with a tetragonal crystal structure in a structurally stable SiOCN matrix *via* the concept of “Nanocomposites Through Chemistry of *Single-Source* Precursors”^37–39^. The basis for this approach comes from the design of a suitable synthetic precursor in which uniform chemical composition is established at molecular scale. Then, precursors are converted in a first pyrolysis step into single-phase amorphous ceramics in which considerable weight loss occur. These are subsequently heat-treated at higher temperature under nitrogen to initiate the crystallization of the nanophase and provide the material with tuned phase composition and nano-/microstructure organization. The proposed new nanocomposites provide new insights not only into the material design by taking advantage of the weight loss occurring during the pyrolysis to form foams but also into the understanding of the photocatalytic behavior not explored before.

## Results and discussion

### Material design

**Figure 1:**
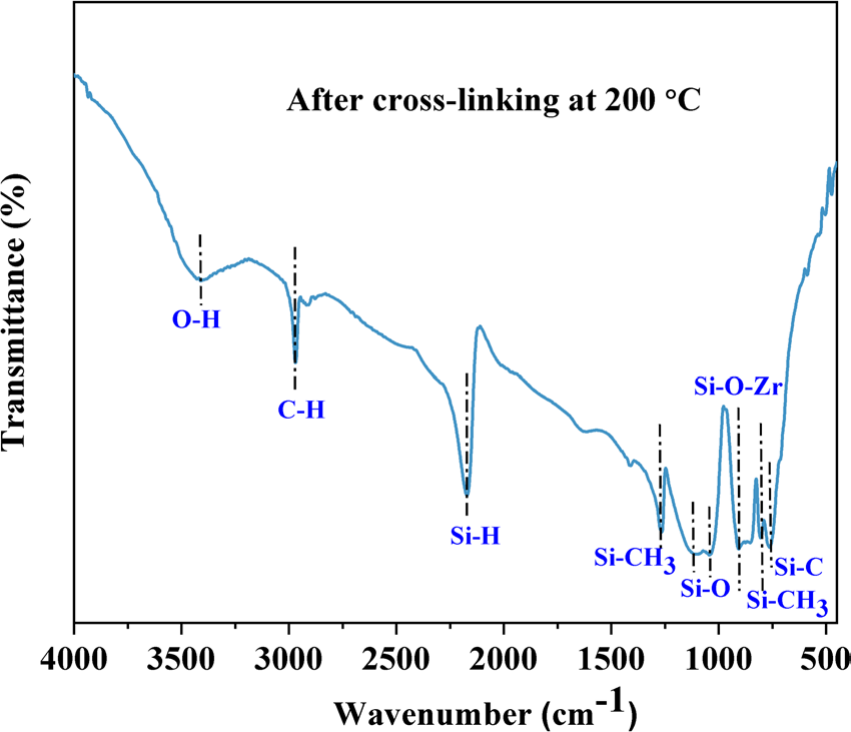
FT-IR spectrum of **Z10-200** cross-linked polymer.

We chose the **Z10** sample to discuss the synthesis process. A representative FT-IR spectrum of **Z10** cross-linked at 200 °C (Fig. 1) was recorded to identify the mechanisms occurring during reaction.

**Figure 2:**
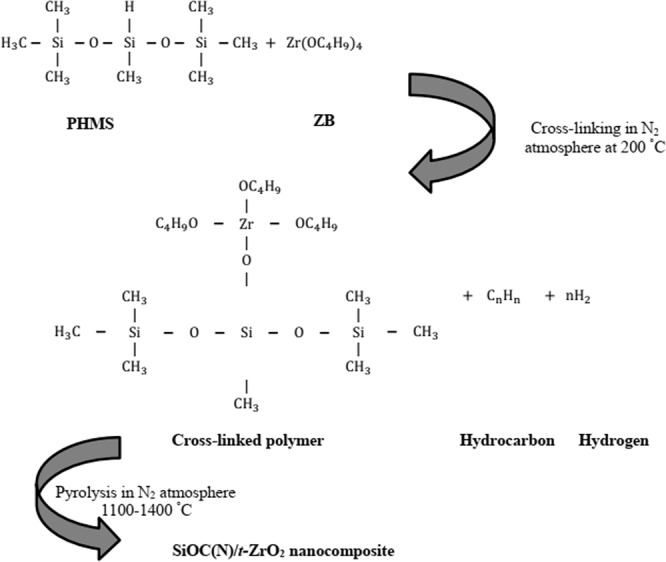
Cross-linking reaction between ZB and PHMS.

The spectrum of the **Z10** sample (Fig. 1) shows peaks at 2965, 2173, 1263 cm^−1^ corresponding to vibrations from ν_as_(C-H), Si-H and ν_s_(Si-CH_3_) stretching respectively^40^. The peaks at 1037 and 1093 cm^−1^ correspond to D units of Si-O vibrations^40^. The broad band at 3418 cm^−1^ is attributed to O-H stretching^40^ and could be due to adsorbed moisture. The band around 900 cm^−1^ corresponds to Si-O-Zr- units^35,36^. The peaks at 755 and 804 cm^−1^ are associated with Si-C and ν_as_(Si-CH_3_) vibrations. The presence of Si-O-Zr units in the polymer gel from FT-IR spectrum indicates the modification of PHMS by ZB. ZB seems to crosslink the siloxane chains through the formation of Si-O-Zr bridges. The cross-linking reaction between ZB and PHMS is exemplified in Fig. 2.

The cross-linked polymer later decomposes to form amorphous SiZrOC. The thermogravimetric analysis of the **Z10** sample (Fig. S1) confirms the completion of ceramization at around 858 °C. The ceramic sample obtained at 1400 °C (for 5 h in nitrogen atmosphere) labeled **Z10-1400** has been fully characterized in parallel to other selected samples obtained with different Zr contents.

**Figure 3:**
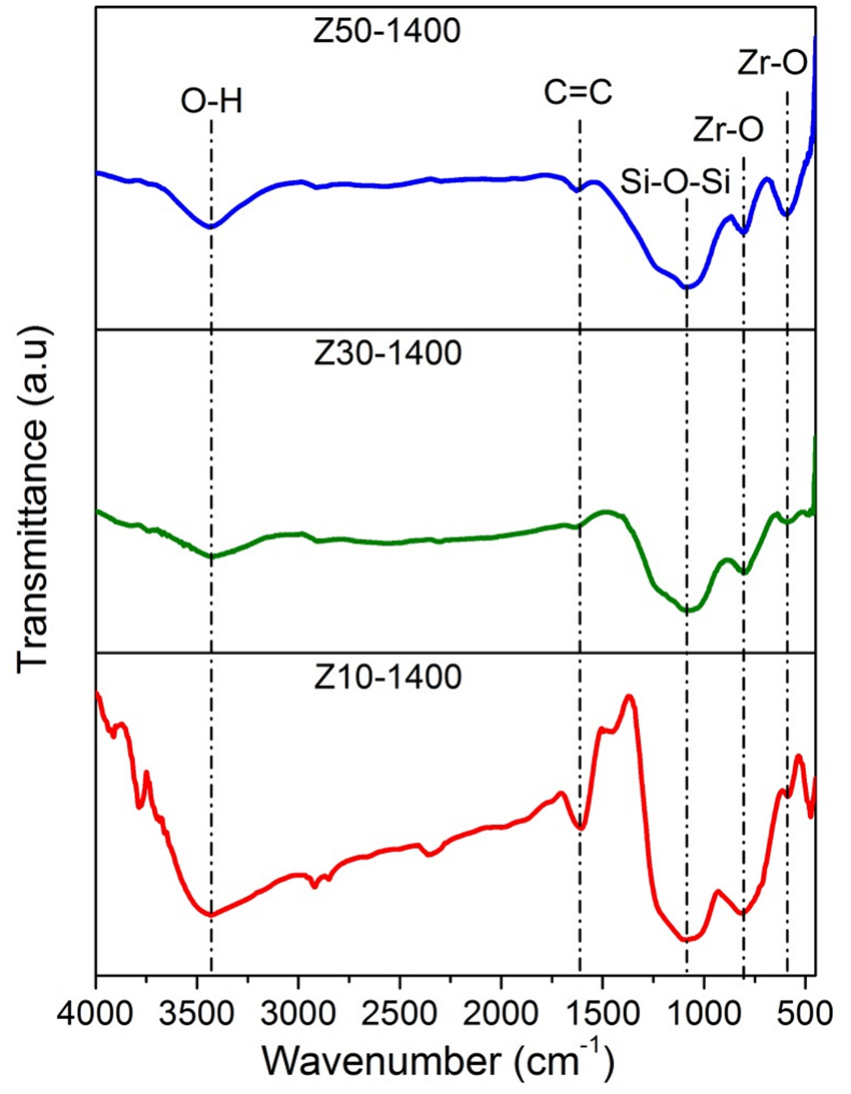
FTIR spectra for different volume fractions of ZB pyrolyzed at 1400 °C.

The FT-IR spectra of **Z10-1400**, **Z30-1400** and **Z50-1400** samples are exemplified in Fig. 3. The band observed at 1094 cm^−1^ corresponds to Si-O-Si stretching vibrations while the last two bands at 787 and 580 cm^−1^ could be associated with the ordered zirconia phase^35^. The overlapping of Si-O-Si and Si-O-Zr peaks results in the broadening of the band at 1094 cm^−1^. As expected, the intensity of Zr-O vibrations becomes sharper with increase in volume percentage of ZB. The band at 1625 cm^−1^ is a result of C = C stretching vibrations. It is also seen that the Si-C band at 755 cm^−1^ observed in the polymer has completely disappeared. Also, this could indicate possible cleavage of Si-C bond and presence of free carbon in the matrix. The broad band at 3400 cm^−1^ observed in all samples comes from O-H functional groups. The intensity of this band increases with increase in the volume fraction of ZB. Earlier reports also suggest an increase of hydroxyl groups with increase in concentration of zirconia^9,15,30^. This suggests that zirconia surface has greater ability to adsorb hydroxyl groups. It is believed that these surface hydroxyl groups interact with the holes generated during photocatalysis.

**Figure 4:**
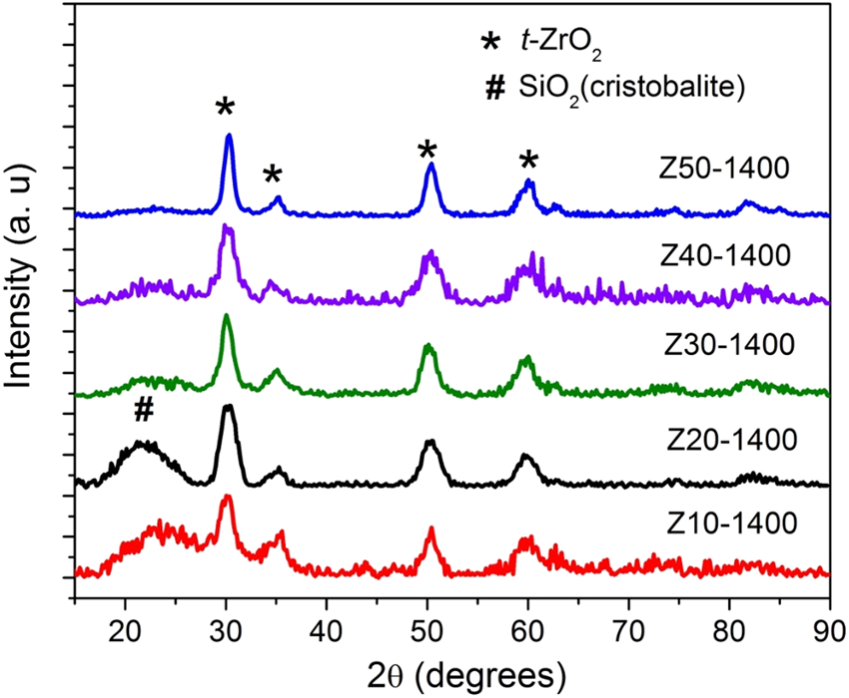
X-ray diffractograms revealing the phase evolution of stabilized *t*-ZrO_2_ as a function of ZB volume fraction at 1400 °C.

X-ray diffractograms (XRD) of the same samples are shown in Fig. 4. In addition, the XRD patterns of **Z20-1400** and **Z40-1400** are reported. The XRD diffractograms are composed of peaks at 30.2, 34.7, 50.2 and 59.7° which correspond to the (011), (110), (112) and (121) planes of tetragonal zirconia, respectively. The corresponding d-spacings are 2.94, 2.55, 1.81 and 1.54 Å. A broad hump observed at around 21° in **Z10-1400** and **Z20-1400** can be assigned to silica and the broadness of the hump indicates the amorphous nature of the phase. This amorphous hump disappears with increase in volume fraction of ZB. The broad amorphous hump weakened for **Z40-1400** and completely disappeared for **Z50-1400**. The decrease/disappearance of SiO_2_ hump could be either due to suppression of the crystallization of silica by ZrO_2_ or the increase in other composition. For further understanding of the effect of pyrolysis temperature on the crystallization behavior, only samples with 10, 30 and 50 volume fractions of ZB were investigated and presented in the manuscript.

**Figure 5:**
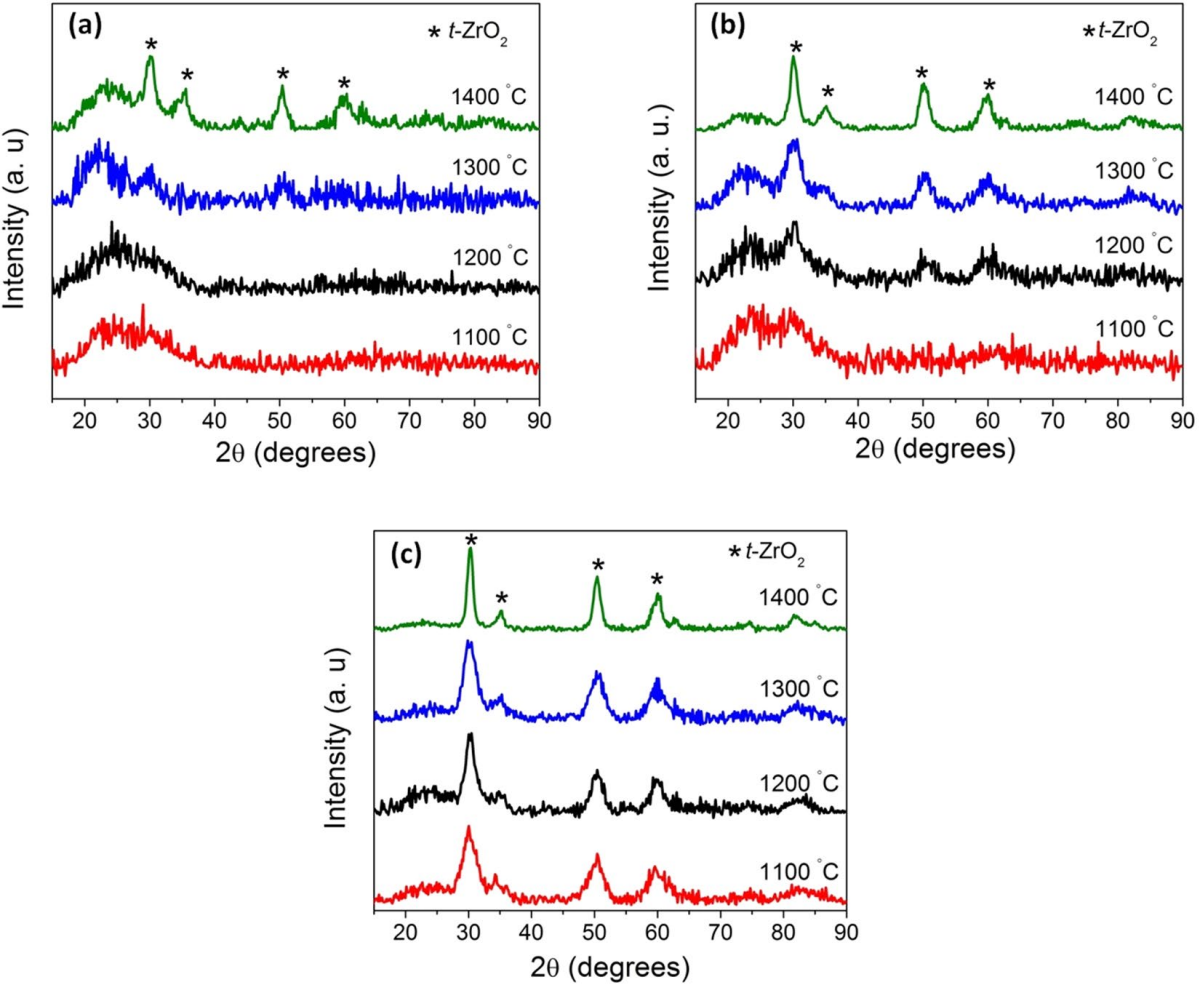
X-ray diffractograms of (**a**) **Z10**, (**b**) **Z30** and (**c**) **Z50** as a function of pyrolysis temperature revealing the phase evolution and nanocrystalline nature of stabilized *t-*ZrO_2_.

Figure 5 shows the XRDs patterns of (a) **Z10**, (b) **Z30** and (c) **Z50** samples pyrolyzed at temperatures ranging from 1100 °C to 1400 °C for 5 h in nitrogen atmosphere. Crystallization of zirconia appears to begin at 1300 °C for **Z10** whereas for **Z30**, the onset of crystallization is further lowered by 100 °C. In the case of **Z50**, well defined peaks corresponding to *t-*ZrO_2_ are observed even in the samples pyrolyzed at 1100 °C. Hence, with the increase in the volume fraction of ZB the onset of crystallization is shifted to lower temperatures as observed by Dire *et al*.^35^.

The crystallite size of *t-*ZrO_2_ was calculated using the Scherrer’s equation as given below.

1 $${\rm{d}}=\frac{k{\rm{\lambda }}}{{\rm{B}}\,\cos \,{\rm{\theta }}}$$

where d stands for the volume-averaged particle/crystallite size, K for the shape factor, λ represents the wavelength of CuK_α_, B refers to the peak broadening at FWHM obtained after correcting for the instrumental broadening and 2 θ corresponds to the Bragg angle in degrees. The calculated crystallite size of **Z10-1400**, **Z30-1400** and **Z50-1400** were found to be 4, 5 and 7 nm respectively.

Srinivasan *et al*.^41^ have reported phase separation of SiOC ceramics at temperatures exceeding 1300 °C. SiOC decomposes into silicon dioxide and silicon carbide. Silicon dioxide further reacts with the excess free carbon and forms silicon carbide. From the XRD (Fig. 5), the matrix was found to be stable till 1400 °C. The retention of the matrix in its amorphous state at 1400 °C is attributed to the presence of zirconia. Ionescu *et al*.^36^ and Dire *et al*.^35^ have shown that presence of zirconia strongly retards the carbothermal decomposition of SiOC. Increase in the zirconia content promotes the cleavage of Si-C bond discouraging SiC crystallization. This is due to the formation of strong interconnected network between the zirconia crystals and D units of silicon in the matrix. Some of the zirconia is still retained as Si-O-Zr units in the matrix as shown in the FT-IR analysis. While the structural and photocatalytic investigations were carried out on all the samples produced, for the sake of clarity and the higher fraction of crystallinity exemplified by **Z50-1400**, the discussion in the manuscript is henceforth restricted to the aforementioned sample.

### Oxygen defects and interstitial carbon

**Figure 6:**
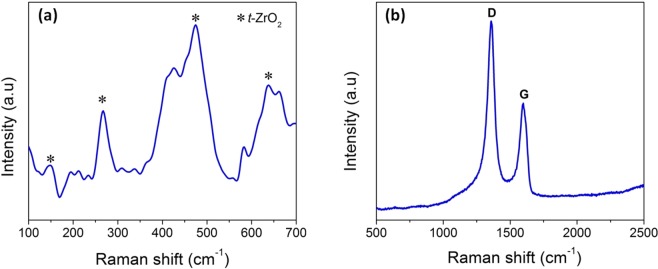
Raman spectra of **Z50-1400** indicating the presence of (**a**) tetragonal zirconia and (**b**) free carbon.

In order further confirm the tetragonal structure of ZrO_2_, Raman spectra of **Z50-1400** was analyzed (Fig. 6). There are six vibrational modes (one A_1g_, three E_g_ and two B_1g_) of tetragonal zirconia that are Raman active^42^ and these modes represent the different symmetries of point groups. The bands observed at 148, 266, 474 and 637 cm^−1^ (Fig. 6a) correspond to the characteristic bands of tetragonal zirconia^43^. The broadness in the bands observed is primarily due to the presence of disordered oxygen sub-lattice. According to Cai *et al*.^44^ disordered lattice activates the Raman scattering from Brillouin zone points. It is this first order process that results in the broadening of the spectrum.

**Figure 7:**
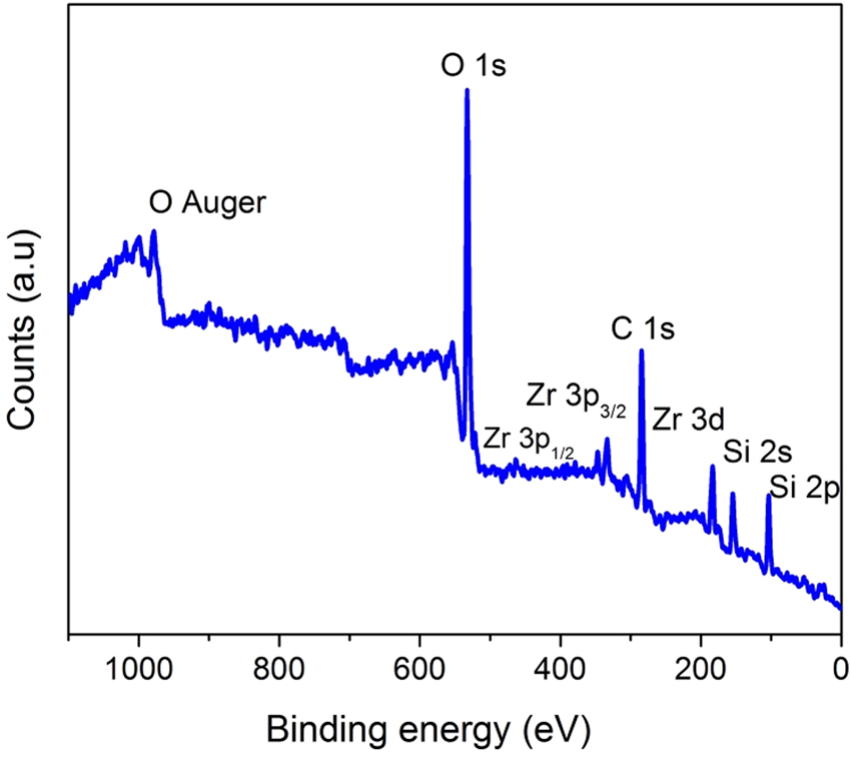
Full survey XPS spectra of **Z50-1400** confirming the presence of silicon, oxygen, carbon, nitrogen and zirconia in the nanocomposite.

**Figure 8:**
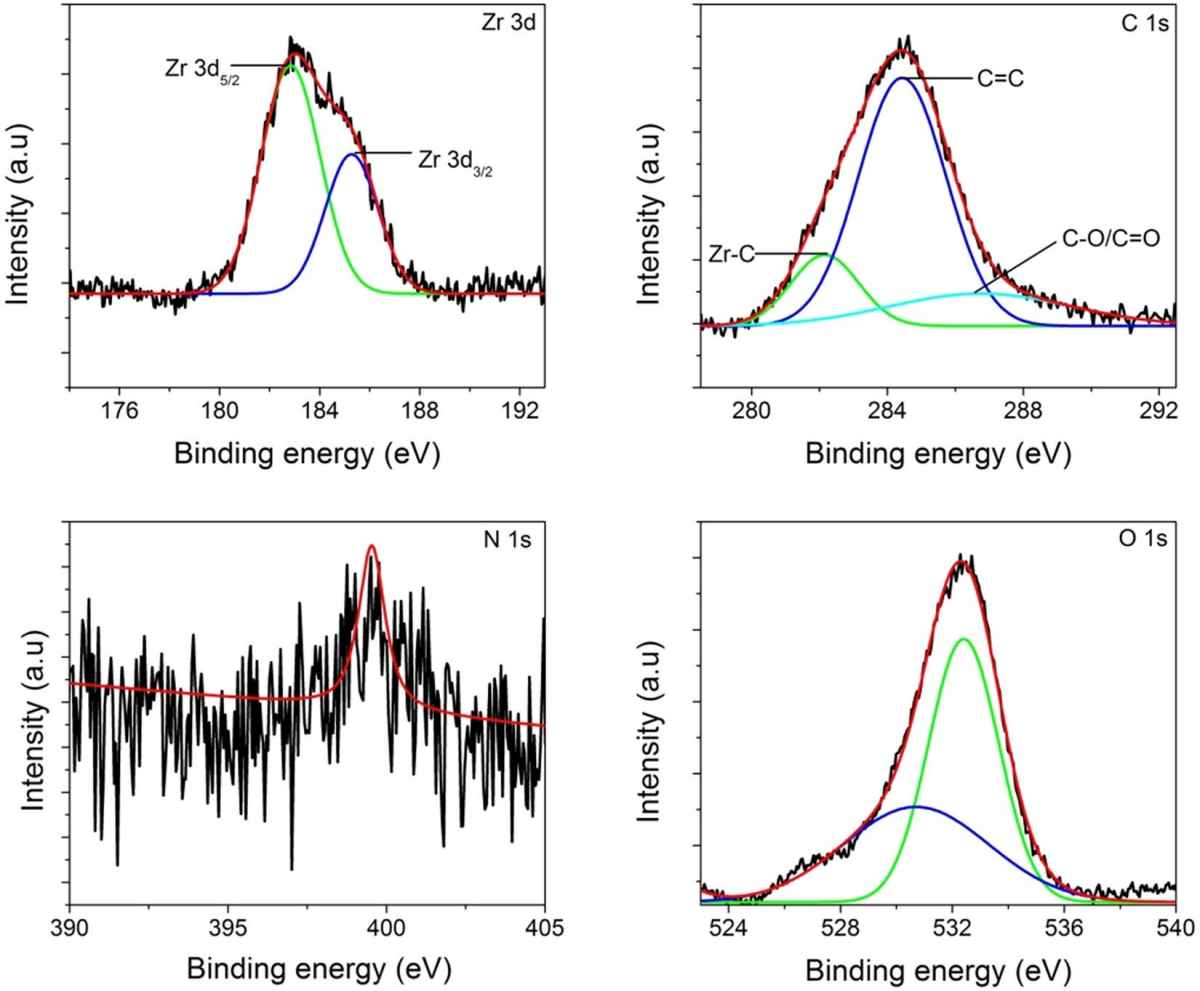
XPS spectra of **Z50-1400** (**a**) Zr 3d (**b**) C 1s (**c**) N 1s and (**d**) O 1s.

The D (1358 cm^−1^) and G (1596 cm^−1^) bands indicate the presence of free carbon in the matrix (Fig. 6b). The intensity ratio of the D band to G band gives an estimate about the defect densities^45^. The I_D_/I_G_ ratio of 1.61 is obtained signifying the presence of large number of defects in carbon which in turn could be attributed to the formation of Zr-O-C bond. As mentioned earlier, increase in the zirconia content promotes the cleavage of Si-C bond resulting in the segregation of free carbon in the matrix. The presence of carbon is shown to increase the absorption of visible light, increase the adsorbing tendency of pollutants, prevent recombination and help in the better transport of electrons from the conduction band to the adsorbed species^33,46^. In order to understand the surface elemental composition as well as the chemical states of zirconium, X-ray photoelectron spectroscopy (XPS) was performed. Figure 7 exhibit the XPS survey of **Z50-1400** which clearly reveals the presence of Si, Zr, O, C and N. In Fig. 8a, the Zr (3d) spectra shows a doublet with Zr (3d_5/2_) and Zr (3d_3/2_) at 182.9 and 185.3 eV respectively which confirms the Zr^4+^ oxidation state in the composite^31^. The undoped ZrO_2_ is reported to exhibit binding energies at 182.2 and 184.5 eV for Zr (3d_5/2_) and Zr (3d_3/2_) respectively which clearly indicates that there is an increase of 0.7 and 0.8 eV from the respective doublets in this study^47^. The shift in doublets could be attributed to lattice distortions. Interestingly, the deconvolution of C (1 s) (Fig. 8b) peak resulted in three peaks at 282.1, 284.4 and 286.7 eV which can be attributed to the presence of Zr-C, C-C and C-O/C=O^48,49^. The peak at 284.4 eV refers to the sp^2^ hybridized carbon^9^. The presence of Zr-C indicates that some of the oxygen atoms in the ZrO_2_ lattice are replaced by carbon atoms (O-Zr-C) creating oxygen vacancies. The presence of zirconia in an amorphous carbon based matrix could also be responsible for the shift in Zr (3d) peaks^9^. A scan explicitly done in the region from 390–405 eV shows an intensity at a binding energy of 399.5 from N 1 s peak (Fig. 8c). The absence of Zr-N peak at 396.8 eV^50^ excludes the possibility of nitrogen doping in ZrO_2_ lattice. Hence the peak at 399.5 eV could be assigned to the bonding of nitrogen to SiOC matrix giving the SiOC(N) label. The deconvolution of O (1 s) region resulted in two peaks at 532.3 and 530.6 eV (Fig. 8d). The peak at 530 eV corresponds to lattice oxygen in ZrO_2_ whereas the peak at 532 eV signifies surface lattice oxygen vacancy^31^. The presence of oxygen vacancies in zirconia nanostructures (as indicated in Raman) is confirmed through XPS analysis. The area under the peak gives a relative oxygen vacancy of 52% (assuming negligible amount of OH adsorption) on the surface of ZrO_2_. The presence of oxygen vacancies is also confirmed through EPR analysis (g value of 1.992)^51^ (Fig. S6). These oxygen vacancies serve as electron traps and prevent recombination of electron and holes during photocatalysis^52^. The peak at 532 eV could also refer to the oxygen in surface adsorbed O-H groups^9,25^. The ability of nanocrystalline zirconia to adsorb hydroxyl groups is highly beneficial for photocatalysis^30^.

### Microstructural and nanostructural features

**Figure 9:**
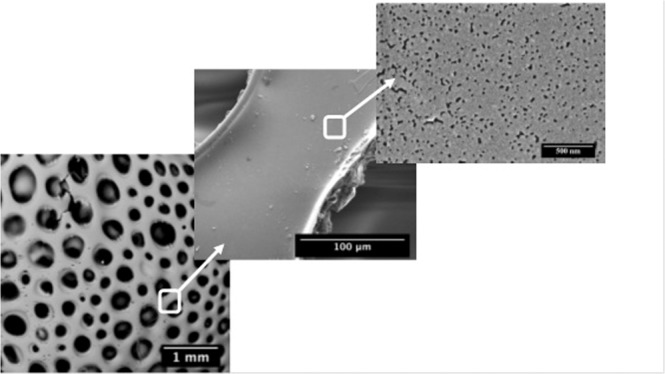
Scanning electron micrographs depicting the hierarchical porous structure of **Z50-1400**.

The SEM images as shown in Fig. 9 show porosity at different length scales with pore sizes ranging from 120 μm down to less than 10 nm, resulting predominantly due to the evolution of oligomers (in the low temperature regime of the pyrolysis) and gases during the pyrolysis. The BET analysis (Fig. S4) revealed the pore diameter to be varying between 4 nm to 150 nm. The porous structure allows adequate contact facilitating the mass transfer between the pollutant fluid and the catalyst, increasing the scattering of light aiding in the photocatalytic process. The pores obtained for **Z10-1400** and **Z30-1400** were non-uniform in nature (Fig. S3).

Figure 9a,b illustrate bright field TEM image of **Z50-1400** nanocomposite. TEM investigation confirms the nanocomposite structural organization of the materials: an uniform distribution of fine spherical zirconia nanocrystals in an amorphous matrix is clearly observed. On an average, around 4–5 nanocrystals of zirconia are present for every 1000 nm^2^ area of the matrix. This means that four active sites are available for photocatalysis per 1000 nm^2^.

**Figure 10:**
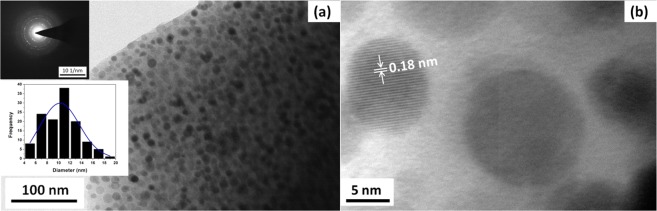
TEM of **Z50-1400** (**a**) indicating the uniform distribution of ZrO_2_ nanocrystals in an amorphous matrix and (**b**) lattice fringes representing *t-*ZrO_2_. Inset image shows SAED pattern and particle size distribution.

The nanocrystallite size distribution obtained from the TEM micrographs is shown in Fig. 10a inset. The histogram was generated from five different micrographs of different volume fractions of ZB. The crystals are of different sizes ranging from 4–20 nm. Maximum number of crystals falls in the range of 6–14 nm. The crystallite sizes obtained from TEM are in agreement with the calculated values using Scherrer’s equation from XRD. It was also observed that the crystallite size of zirconia increases with the volume fraction of ZB, Fig. S5, which substantiate the XRD results.

The SAED pattern recorded for aggregates of crystals in **Z50-1400** is shown in Fig. 10a. The radius of the rings was measured and ratios between them correspond well to the ratios of the d-spacing of (011), (002), (112) and (121) planes of *t-*ZrO_2_ (ICDD-157618). The diffraction from (112) plane is the characteristic feature that distinguishes the tetragonal from cubic phase. The d spacing of 0.18 nm calculated from the lattice fringe spacing (Fig. 10b) corresponds to the (112) plane of the tetragonal structure. It is also evident from the diffraction pattern that matrix is entirely amorphous and there is no crystallization of silica.

### Stabilization of the tetragonal phase

Monoclinic polymorph of zirconia is the most stable crystal structure at room temperature and the tetragonal transformation occurs around 1170 °C. It has been shown that a single strain free zirconia nanocrystal can exist in tetragonal form at room temperature with a critical size less than 10 nm as the surface energy difference overcomes the volume free energy difference^53^. In case of a cluster of crystallites, interfacial energy comes into play increasing the critical size to 33 nm^53^. As shown in Table 1, it is seen that for all types of interfaces the interfacial energy for tetragonal structure is lower than the monoclinic structure^54^.

**Table 1:** Variation in the interfacial energies with crystal structure and type of interface as reported by Garvie^54^.

Type of interface	Monoclinic structure	Tetragonal structure
Incoherent	1.46	1.1
Semi-coherent	0.73	0.55
Coherent	0.29	0.22

A recent study by Jayakumar *et al*.^55^ for pure ZrO_2_ established crystallite size values around 7–20 nm for stable tetragonal phase at room temperature. It is generally observed that at high annealing temperatures (~800–1000 °C), there is considerable size growth resulting in spontaneous transformation to monoclinic structure^46,48^. In the current work, extremely fine crystallite sizes (6–12 nm) are observed even at pyrolysis temperature as high as 1400 °C for extended durations of time most probably because of the presence of a predominantly covalently-bonded matrix surrounding nanocrystals. It is also seen that with the increase in the ZB volume fraction or temperature, the coarsening of *t-*ZrO_2_ is rather minimal, possibly owing to the matrix confinement effect. However, Shukla *et al*.^56^ have shown that with increase in oxygen vacancy concentration, the activation energy for grain growth decreases considerably. Hence, oxygen vacancies seem to contribute in the stabilization of the tetragonal structure of zirconia to maintain its 7-fold coordination. Since pyrolysis of all samples was carried in nitrogen atmosphere at low oxygen partial pressure in the current work, it is expected that the pyrolyzed samples are oxygen deficient which is also confirmed from EPR data (Fig. S6). Also, Xian *et al*.^48^ demonstrated stabilization of tetragonal structure for crystallite sizes as high as ~47 nm and attributed this effect to the presence of carbon. The presence of carbon in the creation of oxygen vacancies can either occur by occupying the interstitial positions of zirconia lattice (as observed in XPS, Fig. 8) or by the reaction between the residual carbon in the matrix and oxygen in the zirconia lattice. The strain energy associated with the SiOC(N) matrix can also strongly retard the transformation to monoclinic structure^35^. Shukla *et al*.^56^ have shown that hydrostatic strain energy values could be as high as 2.1 × 10^8^ J m^−3^, pushing the critical size to a value as high as 200 nm. Hence, the authors believe that the synergistic effect of nanoscale dimensions of ZrO_2_, oxygen vacancies, presence of interstitials such as carbon and nitrogen and the confinement effect of SiOC(N) matrix assist in the retention of tetragonal crystal structure of zirconia at room temperature.

### Bandgap and role of interstitial carbon

**Figure 11:**
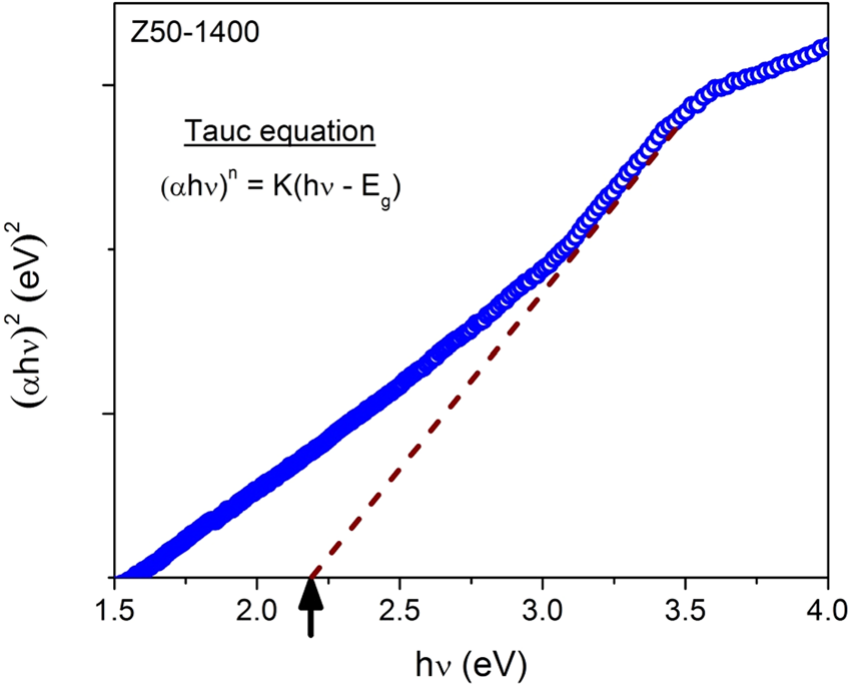
Optical band gap estimation of **Z50-1400** using Tauc plot.

The DRS spectrum of **Z50-1400** is shown in Fig. 11. The band gap (E_g_) was determined by using Tauc Eq. (2)^29^

2 $$\alpha h\nu =K{(h\nu -{E}_{g})}^{n}$$

where, α is the absorption coefficient, hν is the photon energy, K is a constant, and n is either 1/2 for a direct transition or 2 for an indirect transition.

The bang gap was calculated by extrapolating the linear portion of the plot ${(\alpha h\nu )}^{2}$ vs *hv* (assuming ZrO_2_ as a direct semiconductor). The Tauc plot of **Z50-1400** exhibits a band gap of 2.2 eV respectively (Fig. 11).

In general, reported values of band gap for tetragonal structure are around 4-5 eV^9,25,30^. However, the band gap values as low as 3 eV has also been reported by Navio *et al*. for the tetragonal ZrO_2_^57^. The drastic reduction in band gap (2.2 eV) could be attributed to oxygen vacancies and presence of carbon as revealed from XPS (Fig. 7b,d). It is highly probable that carbon enters the interstitial sites of zirconia (O-Zr-C bond) and forms a mid-band gap state between the conduction band and valence band^32^. The presence of oxygen vacancies might also create new energy states between the valence band and conduction band thereby reducing the bang gap and enhancing the visible light absorption^31^.

### Photocatalytic studies

**Figure 12:**
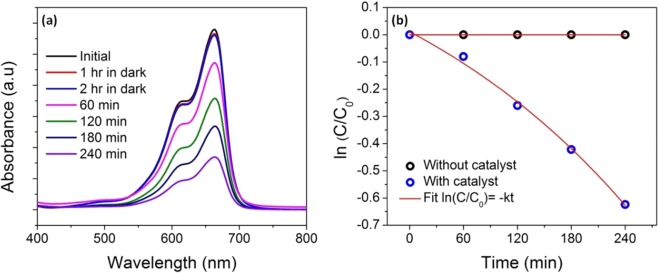
(**a**) UV-vis absorption spectra of methylene blue under visible light exposure and (**b**) ln(C/C_0_) as a function of visible light exposure time. (The catalyst used for degradation study was **Z50-1400**).

The degradation of methylene blue dye after exposure to simulated solar radiation in the presence of catalysts was analyzed using UV-vis spectroscopy. The absorbance pattern of the samples collected from photocatalytic experiments were recorded in the wavelength range of 200–900 nm. The decrease in the concentration of methylene blue dye with time after the adsorption-desorption equilibrium for Z50-1400 is given in Fig. 12a. The absorption value reached a maximum at a wavelength of 663 nm. With the increase in the exposure time absorption peak value at 663 nm decreases, showing the decolorization of the solution in the presence of the catalyst and thus indicating the degradation of the dye. The degradation percentage of methylene blue was calculated according to the following equation^32^.

3 $${\rm{D}}=\frac{{{\rm{C}}}_{{\rm{o}}}-{\rm{C}}}{{{\rm{C}}}_{{\rm{o}}}}\times 100$$

where C_o_ and C are the initial and final concentration in mg/L.

According to the Langmuir-Hinshelwood model^28^ the photocatalytic reaction rate after adsorption-desorption equilibrium is

4 $$\mathrm{ln}(\frac{{\rm{C}}}{{{\rm{C}}}_{{\rm{o}}}})=-\,{\rm{kt}}$$

where C_o_ and C are the concentration at time t = 0 and t = t minutes respectively and k is the reaction rate constant.

It was observed that after 4 h of visible light exposure, 72% of methylene blue was degraded for Z50-1400 nanocomposite. The self-degradation of methylene blue without the presence of catalyst was taken into account and the corrected photodegradation rate constant k (×10^−3^) was found to be 3.2 min^−1^ (Fig. 12b).

### Photocatalysis mechanism

**Figure 13:**
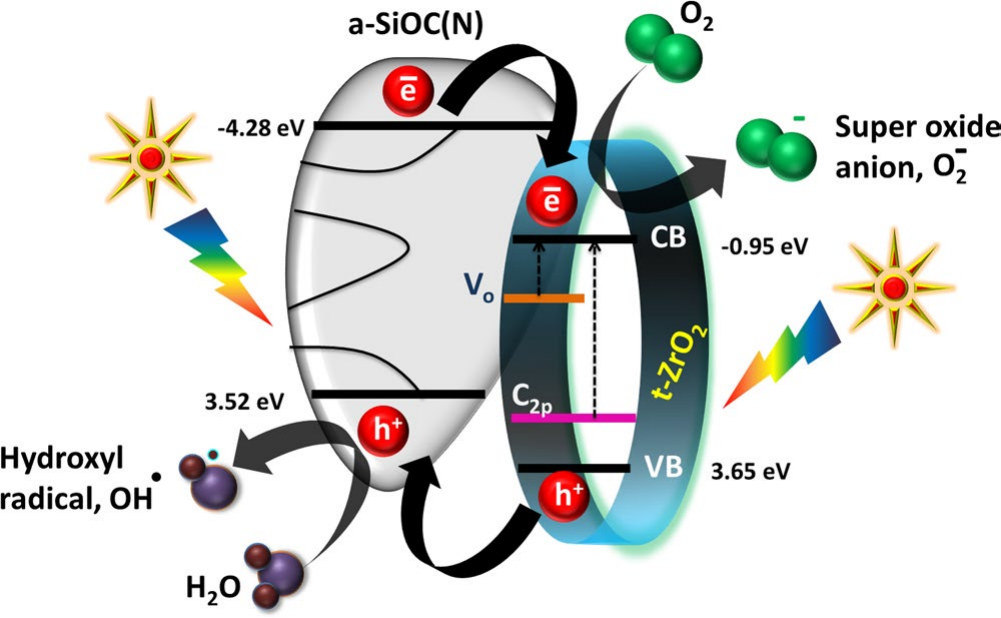
Schematic illustration of the proposed photocatalytic mechanism.

**Figure 14:**
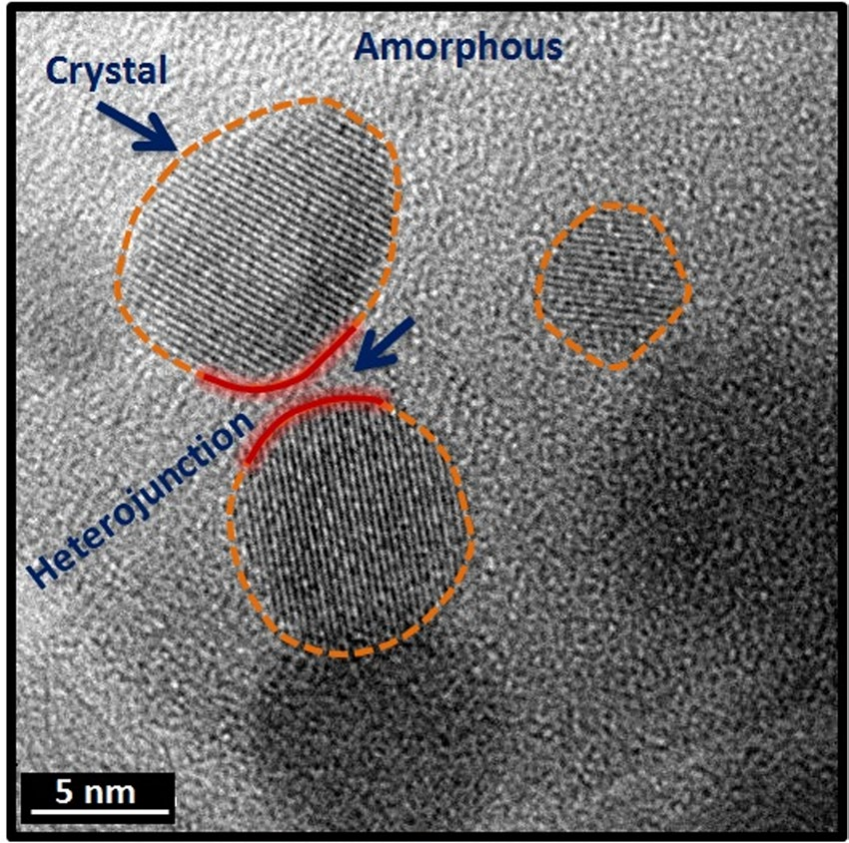
Heterojunction formation between amorphous SiOC(N) and *t-*ZrO_2_.

The absence of long range ordered structure in amorphous silicon oxycarbonitride results in the formation of localized band tails in the valence and conduction bands. The presence of dangling bonds often results in the formation of gap states^58^. The amorphous material is hence expected to possess multiple electron states. The presence of band tails as well as the mid band gap states results in the reduction of band gap in amorphous materials. The formation of heterojunction between an amorphous and crystalline material is a viable approach to improve the photocatalytic efficiency. The interfacial lattice mismatch at the heterojunction between crystalline domains could hinder the transport of the carriers. However, the existence of heterojunctions between amorphous and crystalline material provides an efficient way to separate the photogenerated holes and electrons. This separation combined with the reduced band gap helps in harnessing visible light for photocatalysis^59,60^. Albeit the detrimental contribution *t-*ZrO_2_ towards photocatalytic activity^51^, the formation of heterojunctions as well as the retention of nanodomains of *t-*ZrO_2_ with an average size of 10 nm in an amorphous matrix is believed to enhance the catalytic activity. The degradation mechanism and the heterojunction formation are exemplified in Figs. 13 and 14 respectively. When the nanocomposite is exposed to solar radiation, the electrons are excited from the valence band to the conduction band leaving behind holes. Concomitantly, the photogenerated electrons from the conduction band of amorphous SiOC(N) are transferred to the conduction band of *t-*ZrO_2_. On similar grounds, holes are injected from the valence bands in the opposite direction (i.e., from *t-*ZrO_2_ to amorphous SiOC(N)). This transfer mechanism results in effective charge separation and prevents charge recombination thereby increasing the catalytic degradation efficiency. Further, in order to elucidate the above proposed mechanism, reactive species trapping experiments and EPR measurements were performed (Figs. S7 and S8). The valence and conduction band edges of a-SiOC(N) (Fig. S9) and t-ZrO_2_ has been calculated and included in the supplementary data.

The reaction between surface hydroxyl groups and holes produces hydroxyl radicals which are powerful oxidizers.

6 $${{\rm{OH}}}^{-}+{{\rm{h}}}^{+}\to {{\rm{OH}}}^{\bullet }$$

Subsequently, the captured electrons then react with dissolved oxygen in the polluted water and generate superoxide anions.

7 $${{\rm{O}}}_{2}+{{\rm{e}}}^{-}\to {{\rm{O}}}_{2}^{-}$$

These superoxide anions further react with water molecules generating large number of hydroxyl radicals. These hydroxyl radicals are finally responsible for efficient dye degradation. The decomposition of methylene blue in the presence of hydroxyl radicals is given by Soltani *et al*.^29^

8 $${\rm{MB}}+{\rm{OH}}\cdot \to {{\rm{CO}}}_{2}+{{\rm{H}}}_{2}{\rm{O}}+{{\rm{NH}}}_{4}^{+}+{{\rm{NO}}}_{3}^{-}+{{\rm{SO}}}_{4}^{2-}+{{\rm{Cl}}}^{-}$$

This is the phenomenon responsible for the decrease in the concentration and decolorization of methylene blue and on a large scale reduces the chemical oxygen demand (COD) of textile industry effluents. The synergistic effect of nano *t-*ZrO_2_ and amorphous SiOC(N), oxygen defects, amorphous carbon and porous foamy structure is responsible for the efficient degradation of dye under visible light. The hosting of catalyst particles in an amorphous matrix provides mechanical stability and helps in the easy retrieval from the fluid at the end of dye degradation. This also greatly increases the reusability of catalyst particles.

Dye degradation is a complex process influenced by the nature of dyes^21^, concentration of dye^27^, pH of dye^47^ and catalyst concentration^47^. Hence, a suitable combination of the above variables is necessary for optimum degradation efficiency.

## Conclusions

The crystallization and stabilization of extremely fine nanocrystals (less than 15 nm) of *t-*ZrO_2_ in an amorphous SiOC(N) matrix were confirmed by X-ray diffraction and electron microscopy. The structural characterization depicted the hierarchical porous nature of the nanocomposites. The presence of carbon as well as oxygen vacancies in zirconia lattice confirmed via spectroscopy techniques has resulted in drastic reduction of band gap (2.2 eV). The retention of *t-*ZrO_2_ confined in a matrix (*t-*ZrO_2_/SiOC(N) nanocomposite) exhibited an unusually high photodegradation efficiency of 72% possibly because of the formation of heterojunctions reducing the recombination rate. The synergistic effect of nanoscaled heterojunctions and the presence of carbon and oxygen defects resulted in a paradigm shift pushing the limits of photocatalytic activity zirconia-based nanocomposites from UV to visible region with high degradation efficiency.

## Methods

Zirconium (IV) butoxide (ZB) was mixed with polymethylhydrosiloxane (PHMS) (Sigma Aldrich, Bangalore, India) in varying volume fractions (10–50%) and the mixture was stirred in a magnetic stirrer for about 30 min. In order to facilitate the cross-linking of polymers the stirred mixture was held at 200 °C for 4 h in nitrogen atmosphere. The cross-linked polymers were pyrolyzed in nitrogen atmosphere at temperatures ranging from 1100–1400 °C for 5 h. Nitrogen gas was purged at a flow rate of 2.5 l/min and a constant heating rate of 5 °C/min was maintained during pyrolysis. The samples were cooled down to room temperature and a foamy structure with well-defined pores was obtained. The pyrolyzed samples, hereafter will be referred to as **ZX-Y** throughout the manuscript, where X represents the volume fraction of ZB in PHMS and Y the pyrolysis temperature. The general process for the synthesis of *t-*ZrO_2_/SiOCN nanocomposite is shown in Fig. S1.

The Fourier transform infrared spectra (FT-IR) of ceramic powders were recorded to understand the bonding characteristics (Perkin Elmer Spectrum, USA). The powders were mixed with KBr in the ratio 1:9 and the analysis was done in the transmission mode. X-ray diffractograms (X’PertPRO PANalytical X-ray diffractometer, Netherlands) for all the ceramic samples were obtained in the 2θ range of 10–90° with a step size of 0.008 using Cu K_α_ radiation. The Raman spectroscopy (Labram HR 800 Horiba, USA) measurements were done at an excitation wavelength of 480 nm. The foamy structure of ceramics was imaged using FEI Quanta 200, USA scanning electron microscope (SEM). In order to minimize the charging effect, the samples were sputter coated with gold for 120 s before analysis. The morphology and the lattice fringes of nanocrystals were imaged using Philips CM12 and JEOL 3010 high resolution transmission electron microscopes (HRTEM) respectively at an accelerating voltage of 200 kV. The powder samples were ultrasonicated in ethanol solution and then a few drops were placed on the carbon-coated copper grids. X-ray photoelectron spectroscopy (XPS) was done using SPECS with PHIBOS100 energy analyzer (SPECS GmbH, Germany) to understand the surface elemental composition. Two anodes, Mg and Al act as the X-ray source. The binding energies of the different elements present were analyzed with respect to elemental carbon (284.4 eV) as the standard.

Photocatalytic measurements were carried out in a photoreactor (Heber Scientific, India) with a 500 W tungsten lamp as the light source. In all the measurements the catalyst concentration was kept constant at 50 mg for 100 ml of methylene blue solution of 0.03 mM concentration. The light source was continuously cooled by water circulation through an outer jacket for dissipation of the excessive heat generated. Prior to the exposure, the solution was maintained in dark for two hours under aerated condition to ensure adsorption-desorption equilibrium. The experiments were carried out in visible light for 4 h. At the end of every hour, few ml of solution was collected and centrifuged to remove any solid catalyst particles. The collected samples were further analyzed in UV-vis spectrophotometer (ThermoFischer Scientific, Evolution 220, USA) to study their adsorption characteristics and decolorization behavior. The diffuse reflectance spectra (DRS) were measured with BaSO_4_ as a reference.

## Supplementary information

Supplementary Information
