# Supplementary material for: Design of nanoscaled heterojunctions in precursor-derived t-ZrO2/SiOC(N) nanocomposites: Transgressing the boundaries of catalytic activity from UV to visible light
Source: Sci Rep. 2020 Jan 16;10:430. doi: 10.1038/s41598-019-57394-8 (PMC6965125; doi:10.1038/s41598-019-57394-8)
Supplement: Supplementary file 1 — Supplementary Information [file 41598_2019_57394_MOESM1_ESM.docx]

**Supplementary Information**

**Design of nanoscaled heterojunctions in precursor-derived *t*-ZrO_2_/SiOC(N) nanocomposites: *Transgressing the boundaries of catalytic activity from UV to visible light***

Shakthipriya Bhaskar^1^, Eranezhuth Wasan Awin^1^, K.C. Hari Kumar^1^, Abhijeet Lale^2^, Samuel Bernard^2^, Ravi Kumar^1^

1. Laboratory for High Performance Ceramics, Department of Metallurgical and Materials Engineering, Indian Institute of Technology Madras (IIT Madras), Chennai-600036, India.

Email: [nvrk@iitm.ac.in](mailto:nvrk@iitm.ac.in)

2. Univ. Limoges, CNRS, IRCER, UMR 7315, F-87000 Limoges, France.


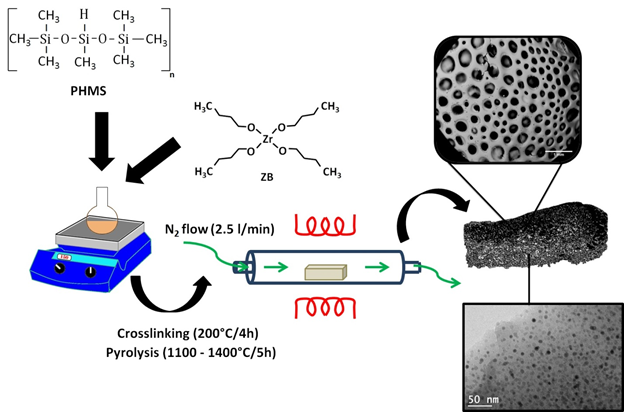


**Figure S1.** Schematic exhibiting the general procedure for the synthesis of the nanocomposite.

- **Thermal analysis**

Thermogravimetric analysis was done to understand the conversion of polymer to ceramic during the pyrolysis process. TG-DTG peaks for the **10%ZB-PHMS** sample is shown in Figure S2. The DTG peak at around 858 °C indicates the completion of ceramization. Thermal analysis of polymer pyrolysis indicates two weight loss zones in the conversion of polymer to ceramic. According to the Weinmann et al.^1^ during the cross-linking stage (200-400 °C) hydrogen and hydrocarbons are released. It is the release of these gaseous byproducts that is responsible for the porosity in the ceramic nanocomposites. The final stage of ceramization (400-800 ^°^C) involves evolution of alcohols, water and cleavage of Si-C bonds leaving behind excess amount of free carbon in the matrix. At the end of thermolysis there is considerable increase in density due to the dramatic volume shrinkage.


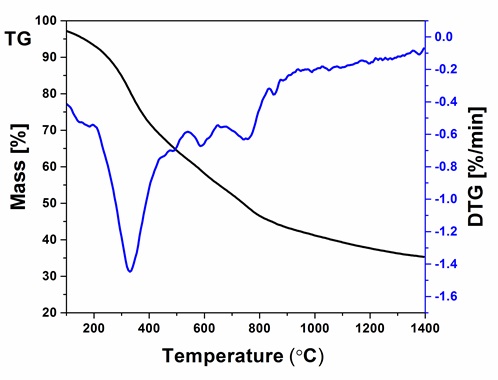


Figure S2. Thermal analysis of **Z10** indicating the mass loss behavior.

- **SEM micrographs**


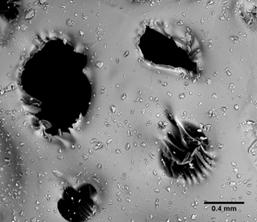

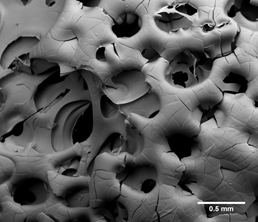


**(a)**

**(b)**

Figure S3. SEM micrograph of (a) **Z10-1400** and (b) **Z30-1400** exemplifying the non-uniform pores.

- **Pore size analysis**


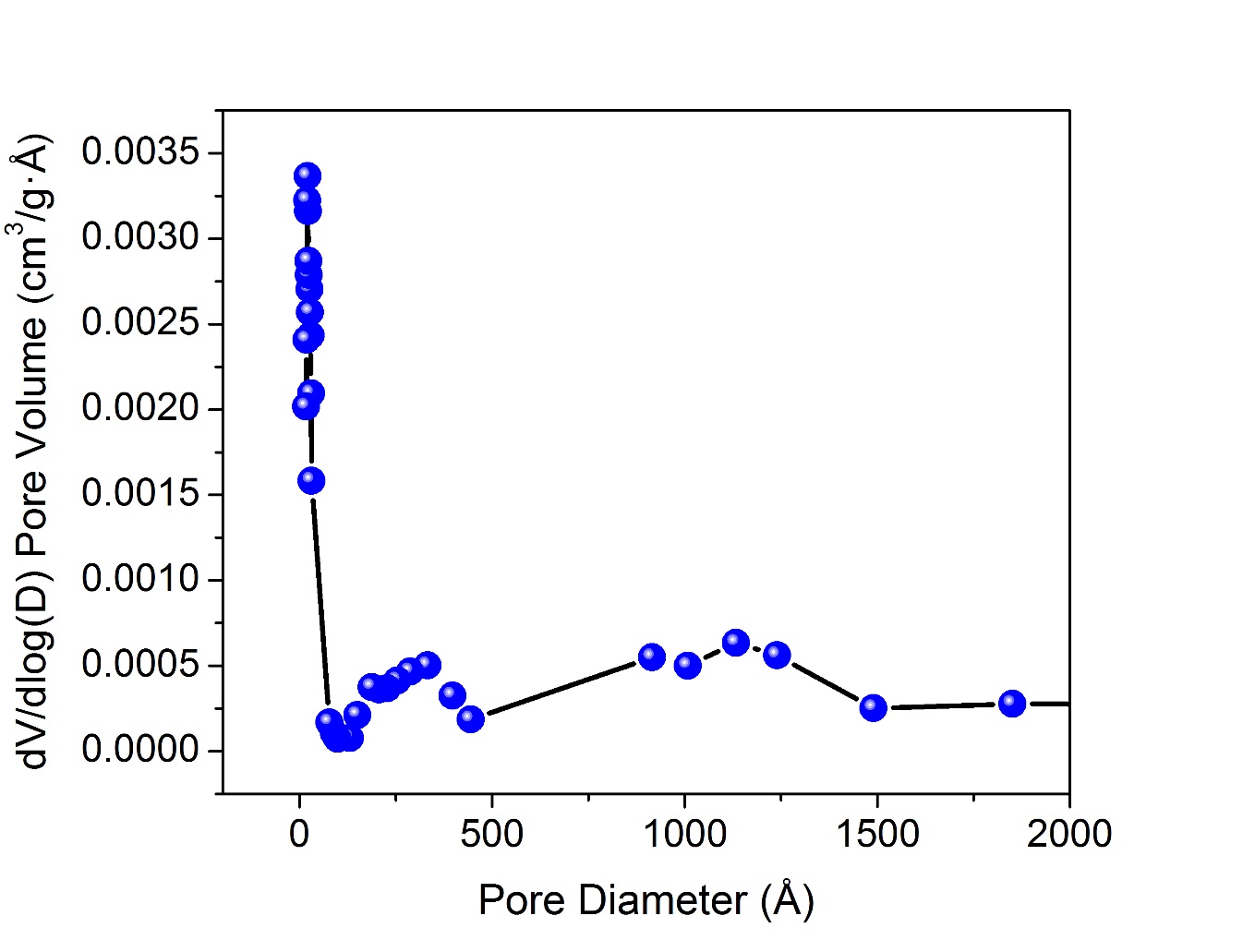


Figure S4. BET pore size distribution

- **TEM** **micrographs**


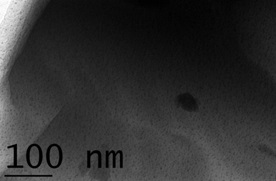


**(a)**


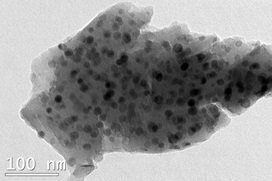


**(b)**

Figure S5. TEM micrograph of **Z10-1400** and **Z30-1400** revealing the variation in crystallite size.

- **Oxygen vacancies**


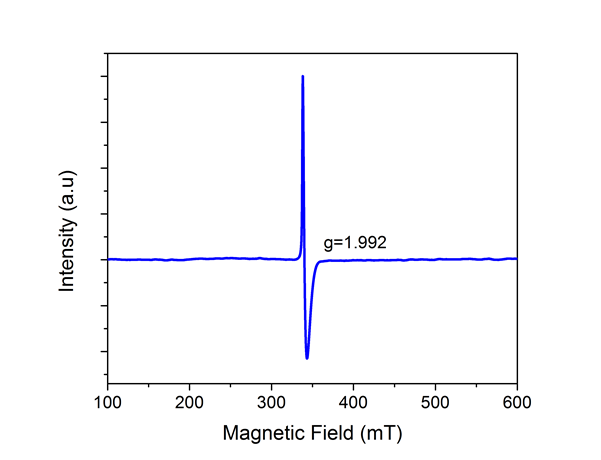


Figure S6. EPR data of **Z50-1400** confirming the presence of oxygen vacancies.

- **Reactive species trapping experiment**

In order to further understand the active species involved in the degradation process, reaction species trapping experiments were performed. 2 mM of Isopropanol (IPA), 0.5 mM of benzoquinone (BQ) and 2 mM of triethanolamine (TEOA) were chosen as quenching agents for $OH^{⦁}$, $O_{2}^{-}$and $h^{+}$respectively. The photocatalytic experiments were performed in identical conditions as indicated in the experimental section and the results are exemplified in Figure S7.

The introduction of IPA and BQ was found to significantly inhibit the degradation process. This implies that the fact that $OH^{⦁}$ and $O_{2}^{-}$ are the major active species during the catalytic activity under visible light illumination. It could be seen that the degradation process was only slightly influenced by the addition of TEOA indicating that $h^{+}$played a secondary role in the catalytic activity. The findings from radical species trapping experiments in turn support the mechanism proposed by the authors where the active species involved in the degradation process are $OH^{⦁}$^.^ and $O_{2}^{-}$.

**
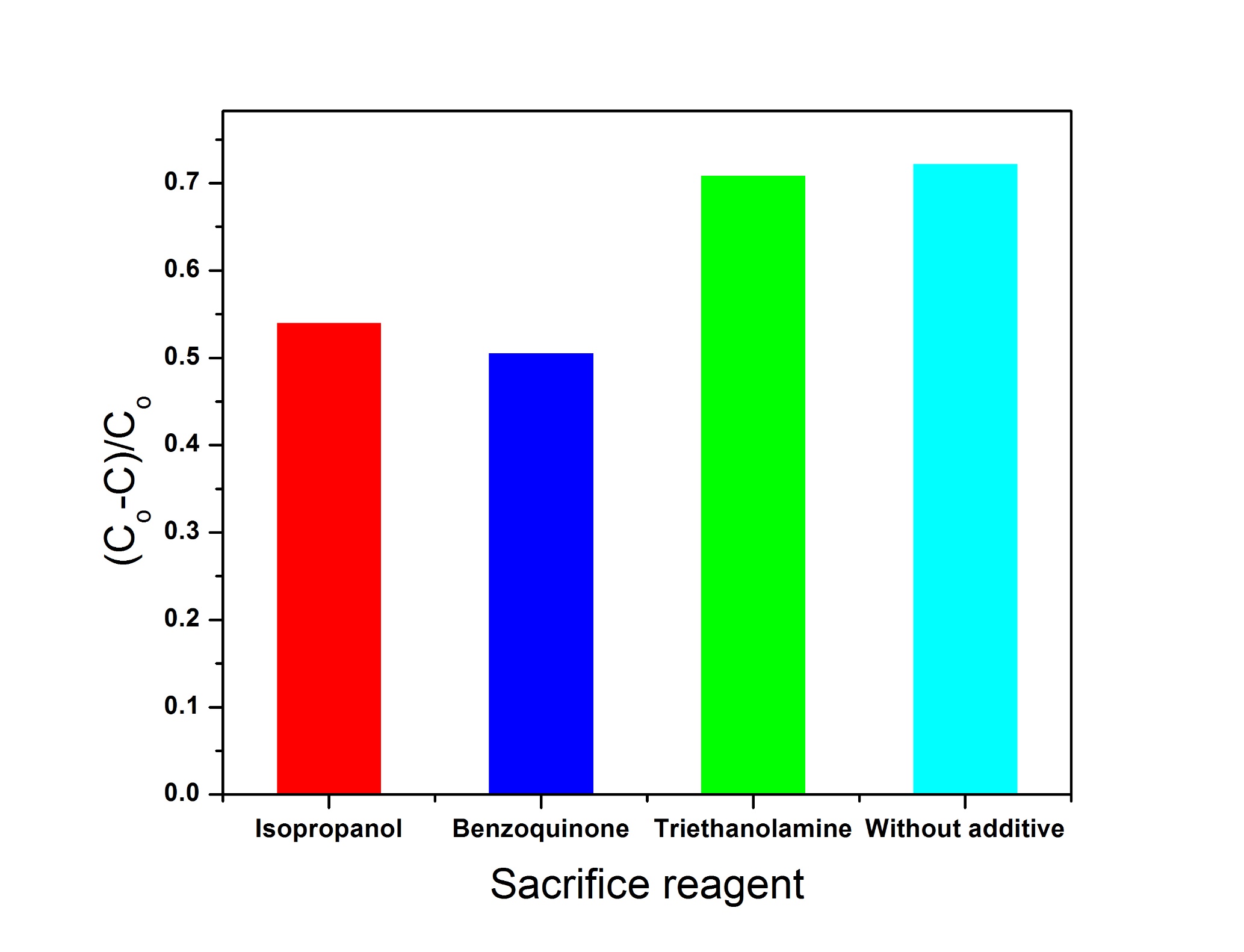
**

Figure S7. Photocatalytic response of **Z50-1400** in the presence of different scavengers under visible light exposure.

- **EPR measurements**

In order to further confirm the production of superoxide anion radicals and hydroxyl radicals, the authors have now performed EPR experiments using 5, 5-dimethyl-1-pyrrolineN-oxide (DMPO) as the spin-trapping agent. The mixtures of t-ZrO_2_/SiOC(N) (1 mg) with DMPO (50 mM) was prepared in water and methanol, targeting ^•^OH and $O_{2}^{-}$ radicals respectively. The mixtures were then irradiated in visible light for 180 s. The enhancement in the intensity of signals after irradiation of visible light in the EPR spectra as shown in Figure S8 confirmed the presence ^•^OH and $O_{2}^{-}$ radicals respectively augmenting the radical species trapping experiments.


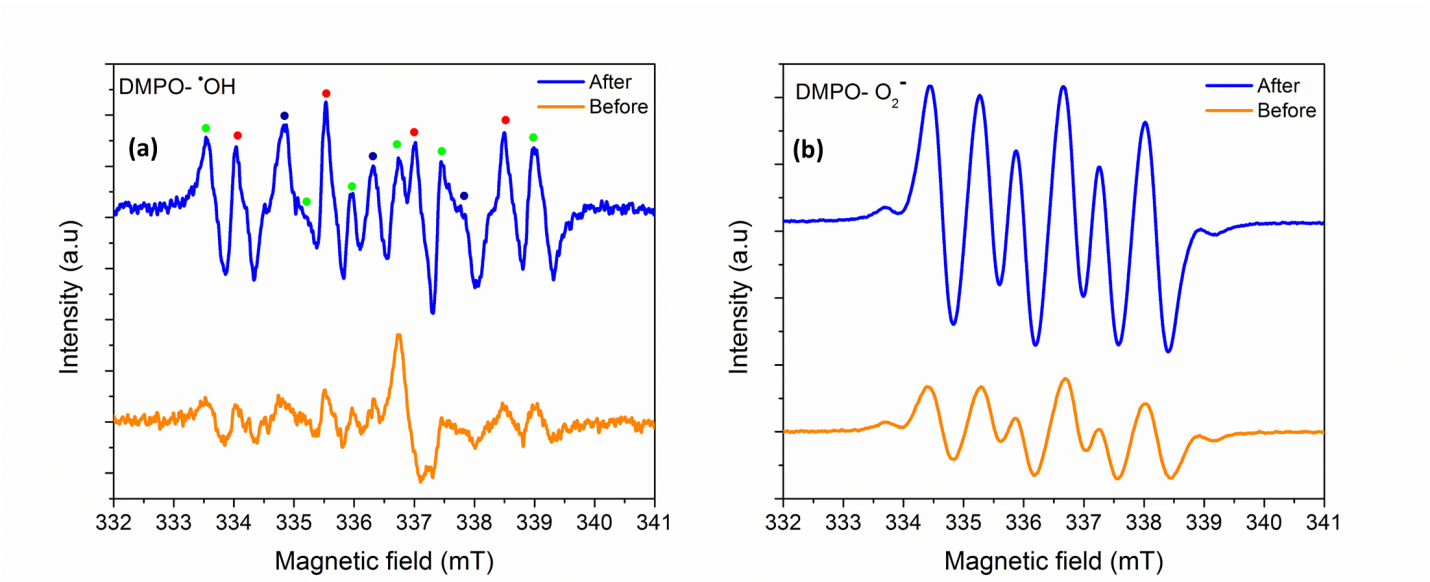


Figure S8. EPR spectra of (a) DMPO-^•^OH and (b) DMPO- $O_{2}^{-}$ of t-ZrO_2_/SiOC(N) nanocomposite under visible-light irradiation upon 180 s. The ^•^OH radical are denoted by red ●, the oxidized DMPO radicals by blue ● and the carbon-centered radicals by green ●.

- **Determination of band structure**

The valence band edge of amorphous SiOC(N) has been determined to be 3.52 eV using XPS as shown in Figure S8. The band gap of amorphous SiOC(N) is taken as 7.8 eV as reported by S. W. King et al. ^2^. The conduction band edge was calculated using the following equation as -4.28 eV.

E_CB_= E_VB_ - E_g_ (1)

where, E_CB_, E_VB_ and E_g_ corresponds to the conduction band edge, valence band edge and band gap of the material.


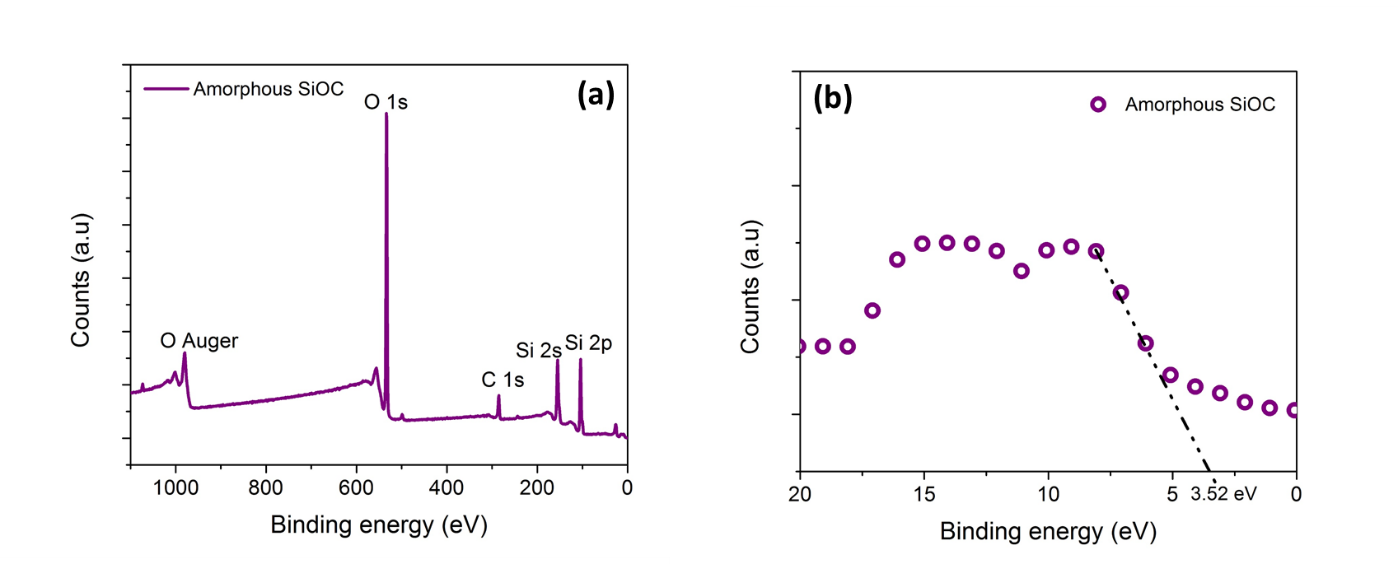


Figure S9. XPS spectra of amorphous SiOC(N) (a) full survey (b) Valence band XPS.

The conduction and valence band edge of t-ZrO_2_ is calculated using the following equation

E_CB_= χ – E_E_ – 0.5 E_g_ (2)

Where, E_E_ is the energy of free electrons vs. hydrogen (4.5 eV) ^3^ and χ is the electronegativity of semiconductor. Eg of t-ZrO_2_ is taken as 4.6 eV as reported by Ajabshir et al. ^4^.

E_CB_ = 5.85 – 4.5 – 0.5 (4.6)

= -0.95 eV

Using the equation (1),

E_VB_ = 3.65 eV

The CB edge potential of a-SiOC(N) (-4.28 eV) and t-ZrO_2_ (-0.95 eV) is more negative than that of O_2_/ $O_{2}^{-}$ (-0.33 eV vs. NHE) ^5^. The VB edge potential of a-SiOC(N) (3.52 eV) and t-ZrO_2_ (3.65 eV) is more positive than that of H_2_O/OH (+2.40 eV vs. NHE) ^6^. Hence both a-SiOC(N) and t-ZrO_2_ straddle the reduction and oxidation potentials.

.

**References**

1. Weinmann, M., Ionescu, E., Riedel, R. & Aldinger, F. *Precursor-Derived Ceramics Ã*. *Handbook of Advanced Ceramics: Materials, Applications, Processing, and Properties* (Elsevier, 2013).

2. King S.W *et al*. Valence and conduction band offsets at low-k a-SiO_x_C_y_:H/a-SiC_x_N_y_:H interfaces. *J. Appl. Phys.* **116**, 113703 (2014)

3. Morrison S. R, *Electrochemistry at Semiconductor and Oxidized Metal Electrode* (Plenum, New York, 1980).

4. Ajabshir S. Z *et al.* A sonochemical-assisted synthesis of pure nanocrystalline

tetragonal zirconium dioxide using tetramethylethylenediamine. *Int. J. Appl. Ceram. Technol.* **11 [4]***,* 654–662 (2014).

5. Deng Y.C., Tang L., Zeng G.M., Wang J. J., Chen. Facile fabrication of a direct Z-scheme Ag_2_CrO_4_/g-C_3_N_4_ photocatalyst with enhanced visible light photocatalytic activity. *J. Mol. Catal. A* **421**, 209–221(2016).

6. Chen F., Yang Q., Sun J., Yao F., Wang S. Enhanced photocatalytic degradation of tetracycline by agi/bivo_4_ heterojunction under visible-light irradiation: mineralization efficiency and mechanism. *A.C.S. Appl, Mater. Interfaces* **8**, 32887–32900 (2016).
